# Probing the existence of non-thermal Terahertz radiation induced changes of the protein solution structure

**DOI:** 10.1038/s41598-021-01774-6

**Published:** 2021-11-16

**Authors:** Martin A. Schroer, Siawosch Schewa, Andrey Yu. Gruzinov, Christian Rönnau, Janine Mia Lahey-Rudolph, Clement E. Blanchet, Till Zickmantel, Young-Hwa Song, Dmitri I. Svergun, Manfred Roessle

**Affiliations:** 1 European Molecular Biology Laboratory (EMBL), Hamburg Outstation C/O DESY, Notkestr. 85, 22607 Hamburg, Germany; 2 University of Applied Sciences Luebeck, Moenkhofer Weg 239, 23562 Luebeck, Germany; 3 Institute of Physics, University of Luebeck, Ratzeburger Allee 160, 23562 Luebeck, Germany; 4 Present Address: Nanoparticle Process Technology, University of Duisburg-Essen, Lotharstr. 1, 47057 Duisburg, Germany

**Keywords:** Biological physics, SAXS, Nanoscale biophysics

## Abstract

During the last decades discussions were taking place on the existence of global, non-thermal structural changes in biological macromolecules induced by Terahertz (THz) radiation. Despite numerous studies, a clear experimental proof of this effect for biological particles in solution is still missing. We developed a setup combining THz-irradiation with small angle X-ray scattering (SAXS), which is a sensitive method for detecting the expected structural changes. We investigated in detail protein systems with different shape morphologies (bovine serum albumin, microtubules), which have been proposed to be susceptible to THz-radiation, under variable parameters (THz wavelength, THz power densities up to 6.8 mW/cm^2^, protein concentrations). None of the studied systems and conditions revealed structural changes detectable by SAXS suggesting that the expected non-thermal THz-induced effects do not lead to alterations of the overall structures, which are revealed by scattering from dissolved macromolecules. This leaves us with the conclusion that, if such effects are present, these are either local or outside of the spectrum and power range covered by the present study.

## Introduction

Within recent years, the use of non-ionizing Terahertz (THz) radiation has found wide applications in the investigation of biological matter^1–5^, e.g., via THz-spectroscopy of protein solutions^6–8^ or THz-based imaging of soft tissue^9^, allowing, due to its sensitivity to the dielectric properties, e.g., non-invasive diagnostics and possible treatment of skin cancer^10–12^. Despite the widened use of THz-radiation, its biological effect is still a topic of recent research^3,5,12–14^.

The possible influence of THz radiation on biological matter can be divided into thermal and non-thermal effects^1,5^. Due to the strong absorption of THz radiation in water, local heating is the major source for structural changes induced in biological materials when using strong THz sources (for power densities of more than several W/cm^2^)^1^. While these conventional thermal effects were studied in greater detail for organisms, tissues, cells and biological macromolecules, the role and impact of the proposed non-thermal effects is rather unexplored and controversial^13^.

Based on the mechanism originally proposed by Fröhlich^16–18^, it is claimed that radiation from the THz part of the electromagnetic spectrum can induce direct coherent excitations within biological macromolecules by coupling to their dipole moments. If the externally supplied energy (e.g. by THz-radiation) to a system of coupled oscillators within a surrounding heat bath (e.g. biological macromolecules in solution) is sufficient, a driven collection of these vibrational oscillators could achieve a highly ordered out-of-equilibrium state, an effect called the Fröhlich condensation^19–22^.

In this coherent state, nearly all vibrational energy is concentrated in the collective motions such that all degrees of freedom of the system oscillate mainly with the same, lowest frequency. As argued by Reimers et al*.*^19^, in biological systems only so-called weak Fröhlich condensation should be feasible. In this case the coupling between the oscillators is weak or even absent, leading to weak incoherent condensates within the biological macromolecules^19^. Even for this weak condensate scenario, however, profound effects on chemical kinetics might be possible. Whether strong, coherent or weak Fröhlich condensation or any such excitations exist in biological systems remains still a matter of debate and subject of research^13^.

In several spectroscopic studies^23–27^, such out-of-equilibrium movements for biological macromolecules have been modelled by large scale vibrational modes. These exhibit theoretical resonance frequencies in the range of 0.3–6.0 THz. Following Fröhlich’s theory, external electromagnetic radiation in the THz-range might therefore excite such collective motions. These types of THz excitations could lead to structural changes of the proteins with the magnitude of several Angströms, if such a mechanism exists, and in turn might lead to shifts of chemical equilibria.

Several studies have recently been focussed on monitoring structural changes of biological macromolecules induced by THz-radiation using different experimental techniques^22,28–34^. Most of these studies, however, only indirectly probed the possibility of such conformational transitions, e.g., by spectroscopic methods^22,28,33^, or after long exposure times of several minutes by observing aggregate formation or disassembly via fluorescence microscopy^30,32,34^, while a direct demonstration of THz-induced excitations of global domain movements is still missing.

Recently, X-ray crystallography revealed THz-induced non-thermal changes in the structure of the proteins lysozyme and trypsin^29,31^. The observed changes were small and not global, but the structural dynamics of native protein molecules in solution is expected to be different from these within the crystal.

In the present work we study the presence or absence of THz-induced structural changes of biological macromolecules in solution. Using small angle X-ray scattering (SAXS) one can probe the solution structure of particles at the nanometre scale. SAXS is particularly sensitive to the overall shape of proteins and changes of the global structure as induced by external perturbations^35–37^, such as temperature^38^, pressure^39^ or light triggering^40^. Using SAXS allows one to probe the existence of THz-induced global structural changes and, in particular, movements of large protein domains as well as shifts of equilibria between different states.

Here, we investigate a set of selected biological macromolecules, which have been proposed to be susceptible to THz-radiation. We chose bovine serum albumin (BSA), a protein frequently used in SAXS studies, which was predicted to be affected by THz-radiation and was in the focus of previous studies^22,28,41^. As another protein system we studied microtubules (MT), which have been frequently suggested to exhibit Fröhlich condensation^42–44^.

**Figure 1:**
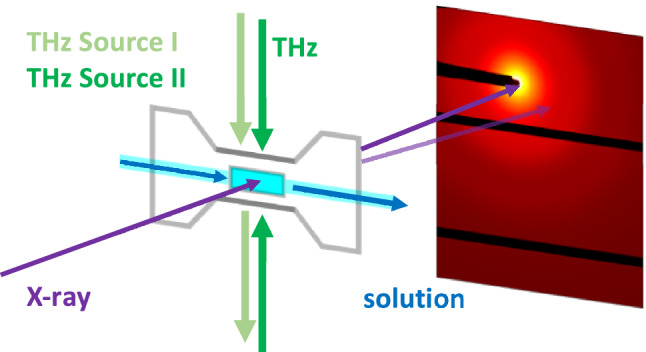
Schematic drawing of the THz-SAXS experiment on protein solutions. The monochromatic X-ray beam (purple arrow) is scattered from the protein solution (blue) flown through a microfluidic cell. Perpendicular to the X-ray beam path and to the flow direction, the solution is irradiated with THz-radiation. THz source I provides the exposure from one side (light green), while source II allows irradiation from both sides (dark green). Two-dimensional SAXS patterns are recorded by a PILATUS 6 M detector (zoom to the central part of the 2D detector image is shown).

Combined THz-SAXS experiments have been performed in several experimental sessions in order to extensively repeat and optimize the measurements. To this end, we have developed a dedicated microfluidic sample environment which was specifically designed for combined THz-SAXS studies^45^. Figure 1 displays a schematic drawing of the experimental design. To probe the effect of different THz-spectra, two types of THz sources were used: (1) An antenna-based source with fixed frequency at 0.5 THz and a power density of Φ = 6.5 mW/cm^2^. (2) A laser based broad-band source with Φ = 0.8 mW/cm^2^. The THz sources used have a relatively low power density compared to other custom-made sources, which results in a small THz radiation dose, in order to reduce the probability for thermal induced effects within the protein solution^1,13^. Moreover, both sources used in this study can be integrated easily to the SAXS setup.

Our results demonstrate that there are no large-scale changes of the structure for the selected proteins studied with the dedicated setup and relatively low power densities. In particular, under none of the THz conditions probed, we observed any changes in the scattering patterns that could reveal collective motions of (sub-)domains. This indicates that possible THz-induced changes in molecular structures have to be considered as local and/or relatively small, not affecting the overall, global protein structure under the studied conditions.

## Results

### THz-SAXS on bovine serum albumin (BSA) solutions

BSA is a well characterized protein, frequently used in SAXS studies and known to be in a monomer–dimer equilibrium in solution^38,46^. THz absorption spectra of solvated BSA show no distinct spectral features, but a dense overlapping spectrum of vibrational modes, which is directly coupled to the protein structure^23^. This suggests that a wide range of THz-frequencies rather than specific wavelengths may initiate structural changes within BSA.

Previous studies reported conformational changes of lyophilized BSA after THz irradiation for several minutes of up to 150 min, as reasoned from optical spectroscopy data^28^. More recently, experimental spectroscopic^22^ and theoretical^41^ studies suggested BSA as a potent target for THz-induced structural changes. Besides these, changes of the monomer–dimer equilibrium by THz-radiation appears possible as the induced oscillator modes could lead to a dissociation of the BSA dimers in solution. An opposite effect, possible enhanced aggregation of BSA induced by THz-irradiation, similar to, e.g., actin filament formation showing an enhanced polymerization rate after 20 min of THz-exposure^30^, should also be detectable by SAXS.

**Figure 2:**
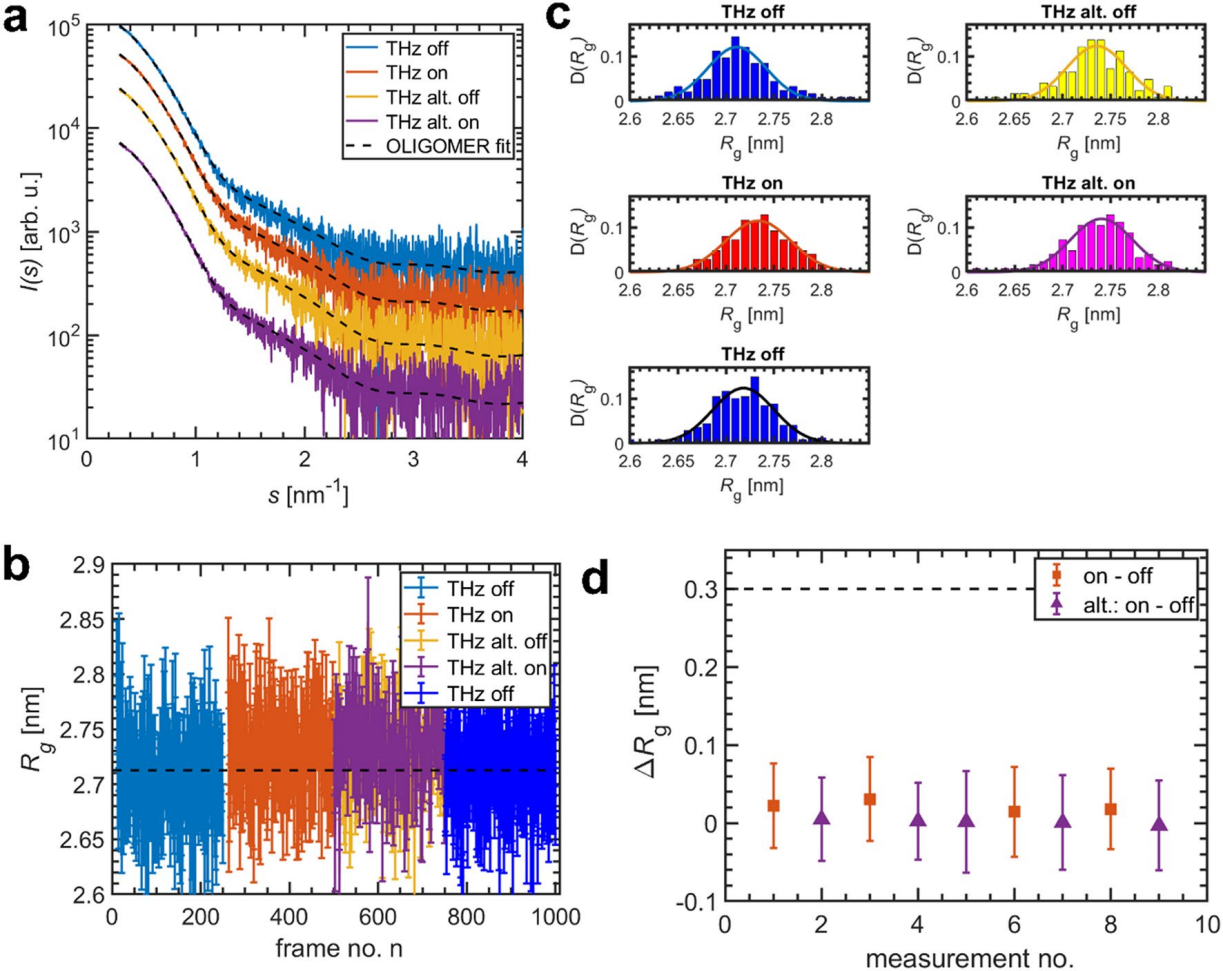
THz-SAXS data for a BSA solution (c = 5.8 mg/mL) at a nominal power density of Φ = 6.5 mW/cm^2^ at 0.5 THz (THz source I). a) Single SAXS curves (exposure time: 0.2 s) out of a sequence of 250 frames without (‘THz off’), with (‘THz on’) and for alternating (‘THz alt. off/on’) THz irradiation. Dashed lines: Fits with an oligomeric mixture of BSA monomers and dimers (obtained with OLIGOMER). All curves were shifted along the logarithmic axis for clarity. (**b**) Radius of gyration R_g_ determined for a sequence of SAXS frames under different THz-irradiation. Dashed line: average R_g_ for ‘THz off’ measurements. (**c**) Normalized histograms of the R_g_-distribution. Solid lines: Gaussian fits. (**d**) Difference of the average radius of gyration ΔR_g_ between THz on- and off -states for several measurements. For alternating radiation, the respective adjacent on/off frames were used. For comparison: Size of the first hydration layer surrounding proteins in solution (dashed line).

These THz-SAXS measurements were performed utilizing different experimental variables to directly probe the effect of THz-radiation on BSA in solution. Figure 2 summarizes the results for a BSA solution (concentration *c* = 5.8 mg/mL) exposed to THz-radiation of nominal power density of Φ = 6.5 mW/cm^2^ at 0.5 THz of THz source I, that results in a deposited energy of ca.75 µJ (see “Methods” for experimental details). For a set of several runs of data collections (each run composed of 250 individual frames), the samples were studied at three different THz states: without THz irradiation (‘THz off’), with full THz irradiation (‘THz on’) and with THz radiation switched off and on between adjacent frames (‘THz alternating off & on’).

Figure 2a depicts exemplary individual SAXS intensity curves *I*(*s*) of one collection run for all four types of conditions. The wave vector reads *s* = 4π/λ sin(θ), where λ denotes the X-ray wavelength and 2θ the scattering angle.

Apparently, THz exposure of Φ = 6.5 mW/cm^2^ has no substantial effect on the curves, as all SAXS profiles exhibit an identical shape (Fig. 2a). This is also reflected when fitting the experimental data by an equilibrium mixture of monomers and dimers (OLIGOMER fit using high resolution models, see “Methods” for details). The whole data set can be well described with this model, that yields volume fractions of the BSA monomer in the range of ${\nu }_{\mathrm{m}}$ = (0.85 ± 0.01). This shows that under all conditions studied, the collected data are well described by a mixture of monomers and dimers with fixed ratio.

As no clear changes induced by 0.5 THz-radiation could be observed in the scattering patterns, a more detailed analysis was applied to quantify whether weak or transient perturbations are present. Therefore, the radius of gyration *R*_g_, which quantifies the effective size of the protein, was determined following the Guinier approximation for small *s*^35,47^, $I\left(s\right)\approx I\left(0\right)\,\,\mathrm{exp}\left(-\frac{{s}^{2}{R}_{\mathrm{g}}^{2}}{3}\right).$ Here, *I*(*0*) denotes the forward scattering. Employing the Guinier approximation, *R*_g_ serves as a probe of global changes of the protein structure from the lowest angles of the SAXS profile (*s* < 1.3/R_g_).

Figure 2b) shows the radius of gyration *R*_g_ extracted for each individual frame *n* for the different states of THz exposure. Besides the frame-to-frame variations within a single run, a very weak increase of *R*_g_ for the THz on-state is present (compared with the mean *R*_g_^off^, dashed line), which seems to disappear upon switching off the radiation. When looking on the respective radius of gyration histograms (Fig. 2c), a maximum of the normalized *R*_g_-distribution for the off-state is at <${R}_{\mathrm{g}}^{\mathrm{off}}$>  = 2.71 nm, which for the on-state is slightly increased, <${R}_{\mathrm{g}}^{\mathrm{on}}$ >  = 2.73 nm. The value changes back close to the initial *R*_g_ upon switching off the THz-radiation. The width of the *R*_g_-distribution (Fig. 2c) does not change indicating that the sample homogeneity is not influenced by THz.

However, the differences Δ*R*_g_ = <${R}_{\mathrm{g}}^{\mathrm{on}}$ > – <${R}_{\mathrm{g}}^{\mathrm{off}}$  > between on and off-state are extremely tiny (~ 20 pm) and are, for example, smaller than the size of the first hydration shell of proteins *d*_Hyd_ = 0.3 nm^48^ (Fig. 2d). For alternating irradiation, which is performed to rule out any systematic changes between two collection runs, there is no change of the effective size. Note that for these, pairs of adjacent on/off frames were used. This suggests that there are either no THz-induced changes or that the very weak changes are still present in the off-state, with a lifetime of more than 200 ms, the exposure time for a single frame. Similar observations hold for the other parameters determined, i.e., the monomer volume fraction ${\nu }_{m}$ and the discrepancy ${\chi }_{n,n+1}^{2}$ (see also §SI-1).

To further explore, whether THz-radiation in a different frequency range can induce structural changes for BSA, we used a different THz-source and performed THz-SAXS measurements in several experimental runs. This THz source II creates pulses at 100 MHz repetition rate of a broad spectral range (0.1–6.0 THz), with an averaged power density of Φ = 0.8 mW/cm^2^ and a deposed energy of ~ 10 µJ. For this setup, the data collection strategy was modified: instead of long collection runs of several hundred individual frames, a short collection of 50 frames was recorded and directly averaged.

**Figure 3:**
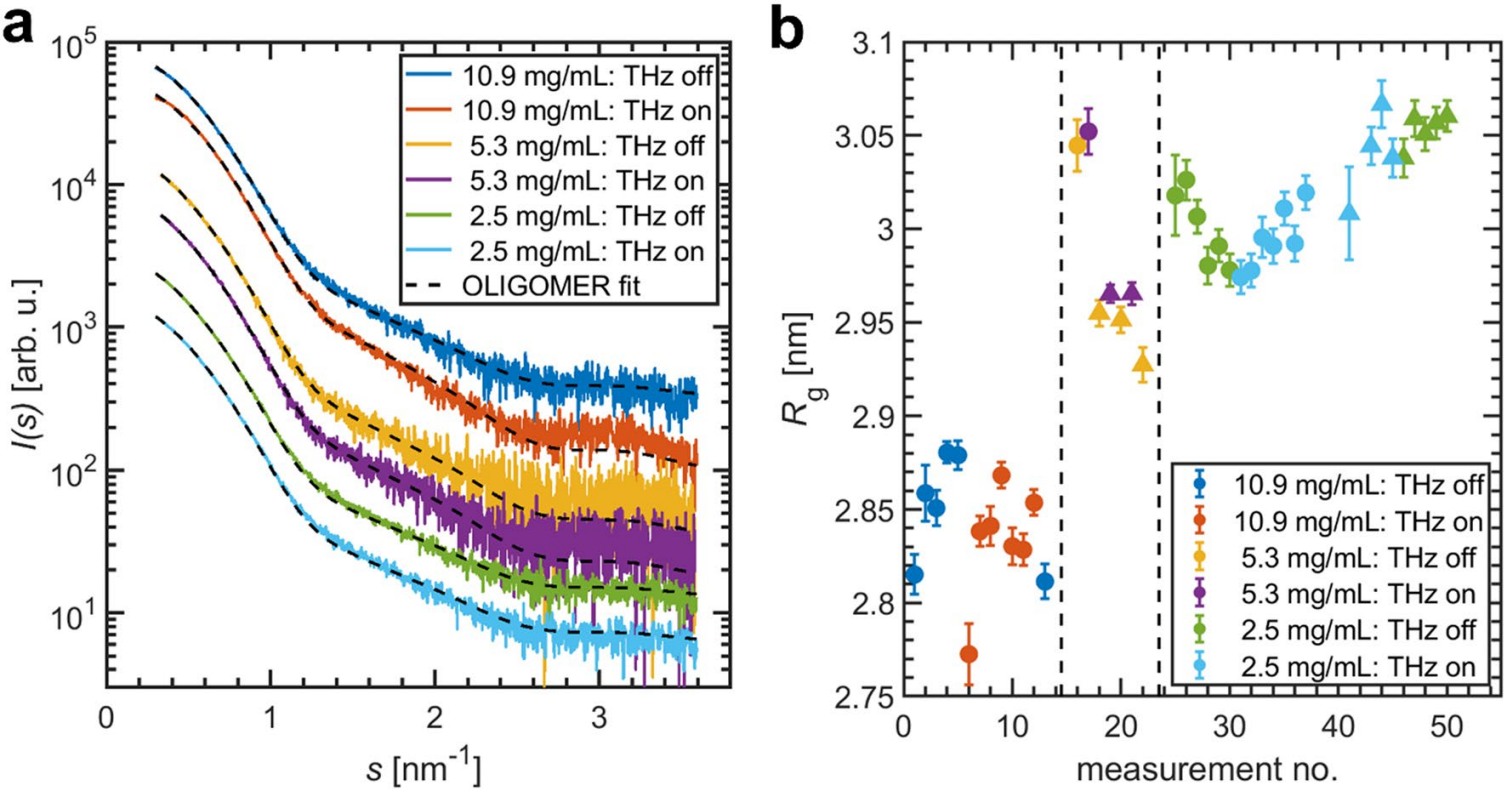
THz-SAXS data for BSA solutions of different concentrations exposed to an average nominal power density of 0.8 mW/cm^2^ (THz source II). (**a**) SAXS profiles and OLIGOMER fits. Data for *c*  =  5.3 mg/mL were collected in different experimental sessions. The displayed curves are shifted along the logarithmic axis. (**b**) Radius of gyration for different repeated data collections. Different symbols mark different sample batches.

Figure 3a shows representative SAXS curves for BSA solutions of different concentrations collected during several experimental sessions using THz source II. For a concentration *c* = 5.3 mg/mL, which closely matches the concentration used with THz source I (data above), the radius of gyration of the on-state is again only slightly larger than for the off-state (Fig. 3b). Using a different batch of the same sample, a similar effect is present, however, the *R*_g_-variation between the two batches is actually higher than the very weak changes observed for THz-irradiation. A similar effect is observed for the monomer fraction (see SI-Fig. 3). This suggests that also a different THz-spectrum does not have any visible effects on the structure or oligomeric equilibrium mixture of BSA.

In order to test if the protein concentration has any effect on the THz interaction, BSA solutions of a lower (*c* = 2.5 mg/mL) and higher (*c* = 10.9 mg/mL) concentration were studied. As the absorption of THz radiation is predominantly due to water^49^, increasing the protein concentration actually reduces the absorption losses^23,50^ and should, in principle, allow to excite a larger number of protein molecules. Furthermore, following the Fröhlich theory, one can speculate that changing the particle number density might have an influence on the coupling within the Fröhlich condensate as well.

While changing the concentration does have an influence on the radius of gyration (Fig. 3b; ~ 0.2 nm) and the monomer fraction (SI-Fig. 3c), there is no difference for THz-exposure of more than 0.05 nm. For all concentrations studied, the SAXS profiles can be described as a monomer–dimer mixture (Fig. 3a). We attribute the slightly smaller *R*_g_ for the highest BSA concentration to the presence of a weak structure factor contribution due to the repulsive interparticle interactions, an observation common for concentrated protein solutions^38,51^. For smaller concentrations (*c* = 2.5 mg/mL), the *R*_g_-values of different runs show variations, but do not reveal any clear dependence on THz-radiation.

**Figure 4:**
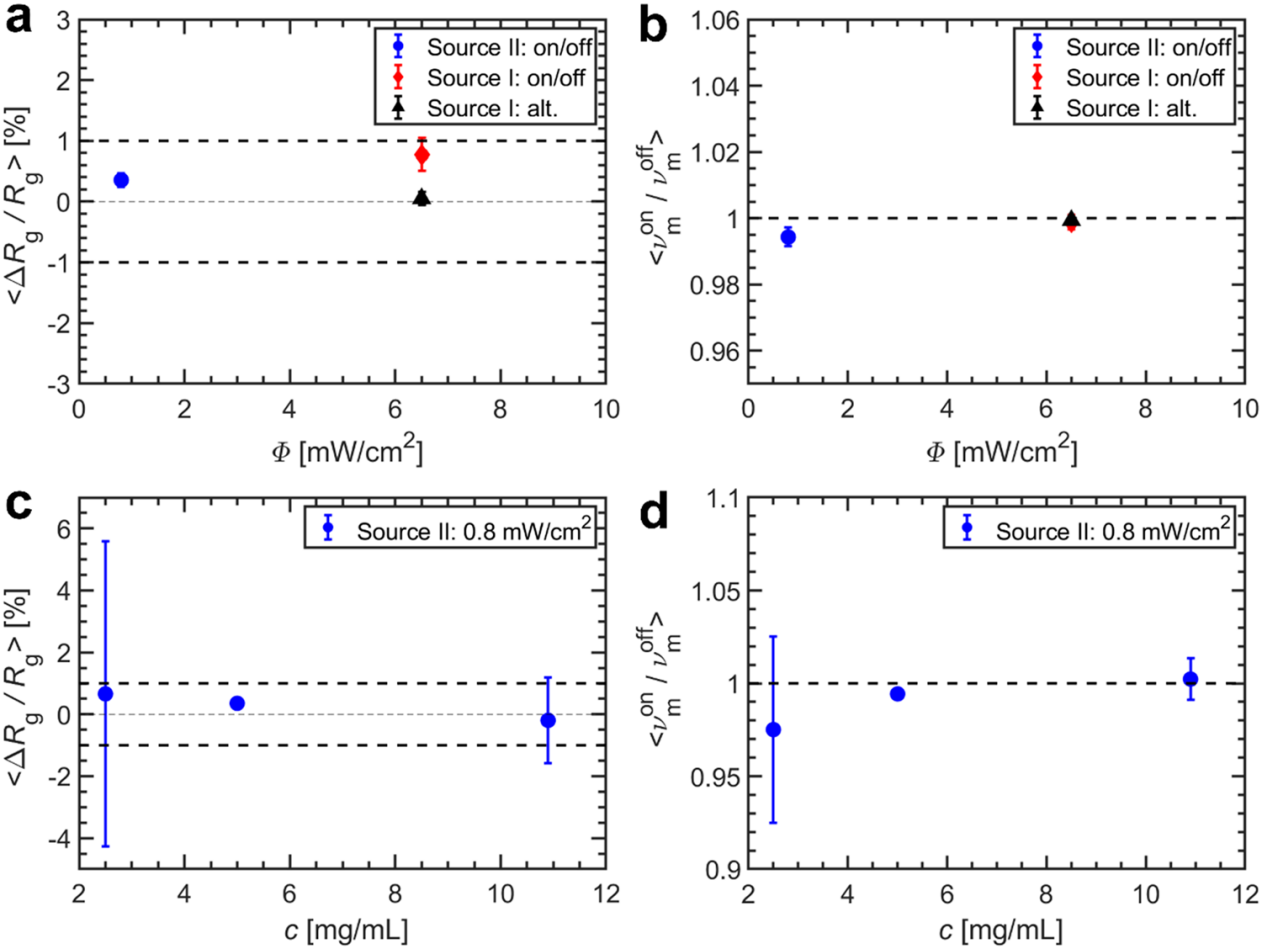
Structural parameters of BSA under THz-irradiation. (**a**) Average ΔR_g_/R_g_ as a function of the nominal radiation power density Φ (for a protein concentration c ≈ 5 mg/mL). Deviations of ± 1% are marked by bold dashed lines. (**b**) Average monomer fraction ${\upnu }_{\mathrm{m}}^{\mathrm{on}}/{\upnu }_{\mathrm{m}}^{\mathrm{off}}$ as a function of Φ. The ratio of ‘1’ is marked by bold dashed lines (**c**) < ΔR_g_/R_g_ > and (**d**) <${\upnu }_{\mathrm{m}}^{\mathrm{on}}/{\upnu }_{\mathrm{m}}^{\mathrm{off}}$ > for different protein concentrations.

To give an estimate for the maximum extent of the changes for BSA in solution based on the experimental SAXS data, the average relative difference of the radius of gyration, < Δ*R*_g_/*R*_g_>  =  <(${R}_{\mathrm{g}}^{\mathrm{on}}$—${R}_{\mathrm{g}}^{\mathrm{off}}$)/${R}_{\mathrm{g}}^{\mathrm{off}}$>, and the average ratio of monomer volume fraction for ‘on’ and ‘off’ conditions, < ${\nu }_{\mathrm{m}}^{\mathrm{on}}/{\nu }_{\mathrm{m}}^{\mathrm{off}}$>, were computed. Figure 4 depicts these parameters under the different experimental conditions varied in this study, summarizing the THz-SAXS results for the BSA solutions.

For the power density and THz-spectra achievable with the two sources used, no significant changes are observed. For THz source I (Φ = 6.5 mW/cm^2^), a very small change of < Δ*R*_g_/*R*_g_>  = (0.8 ± 0.3) % can be found. For alternating irradiation, using adjacent frames, there is no change of the effective size. For THz source II (Φ = 0.8 mW/cm^2^), changes of protein size are even smaller than for source I. A direct correlation to the THz power density cannot be made due to the weakness of the effect. The average monomer ratio ${<\nu }_{\mathrm{m}}^{\mathrm{on}}/{\nu }_{\mathrm{m}}^{\mathrm{off}}>$ is also nearly unchanged for both setups (Fig. 4b). Similar to the intensity and frequency range of the THz source, variation of the protein concentration did not lead to changes of the measured parameters by exceeding 1% (Fig. 4c, d). No detectable changes were further found for the data collected from BSA samples, which have been pre-exposed as powders and then dissolved for SAXS measurements (see §SI-2).

### THz-SAXS on microtubules (MT)

MT is a protein system of very different morphology from BSA solutions, and MTs were frequently postulated and studied to exhibit Fröhlich condensation^42–44^. MT are tubular protein complexes of tubulin and a major component of the eukaryotic cytoskeleton, involved in a range of functions including intracellular trafficking, cell division, and the establishment and maintenance of cell shape^52^. MT are hollow protein nanotubes, comprised of globular dimeric tubulin subunits aligned end-to-end to form linear protofilaments, which interact laterally to form a hollow MT cylinder of up to micron-length^53^. Due to this cylindrical assembly of polar subunits, there have been several speculations in the past, that MTs could serve as waveguides or cylindrical resonators for electro-magnetic radiation^42,54,55^. Especially thanks to their relatively high elasticity^56^, coupling of radiation might induce longitudinal vibrations in MTs^42,43^.

Theoretical computations suggested that MTs can exhibit vibrations within the MHz–GHz range^43,44^, and experimental observations were reported that electro-magnetic radiation in this range can actually lead to MT self-assembly^57^, and recently also disassembly was found^34^. While MHz excitation would refer to a large-scale excitation of the entire, micro-sized MT, additional excitation on shorter length scales, in particular in the THz-regime, have also been proposed to be possible^42,57^.

The presence of longitudinal vibrational waves might induce structural variations across the MT compared to the non-excited state. Furthermore, such excitations could affect the equilibrium between MT singlets and multiplets, e.g. doublets. To probe if global structural changes can be excited, we performed THz-SAXS measurements on MT solutions in two independent experimental sessions. THz source II was used with a one-beam configuration, in which only one emitter was used (Φ = 0.4 mW/cm^2^, one sided irradiation similar to THz source I) and a two-beam configuration, with two emitters doubling up the power densities to Φ = 0.8 mW/cm^2^ by irradiation from both sides.

**Figure 5:**
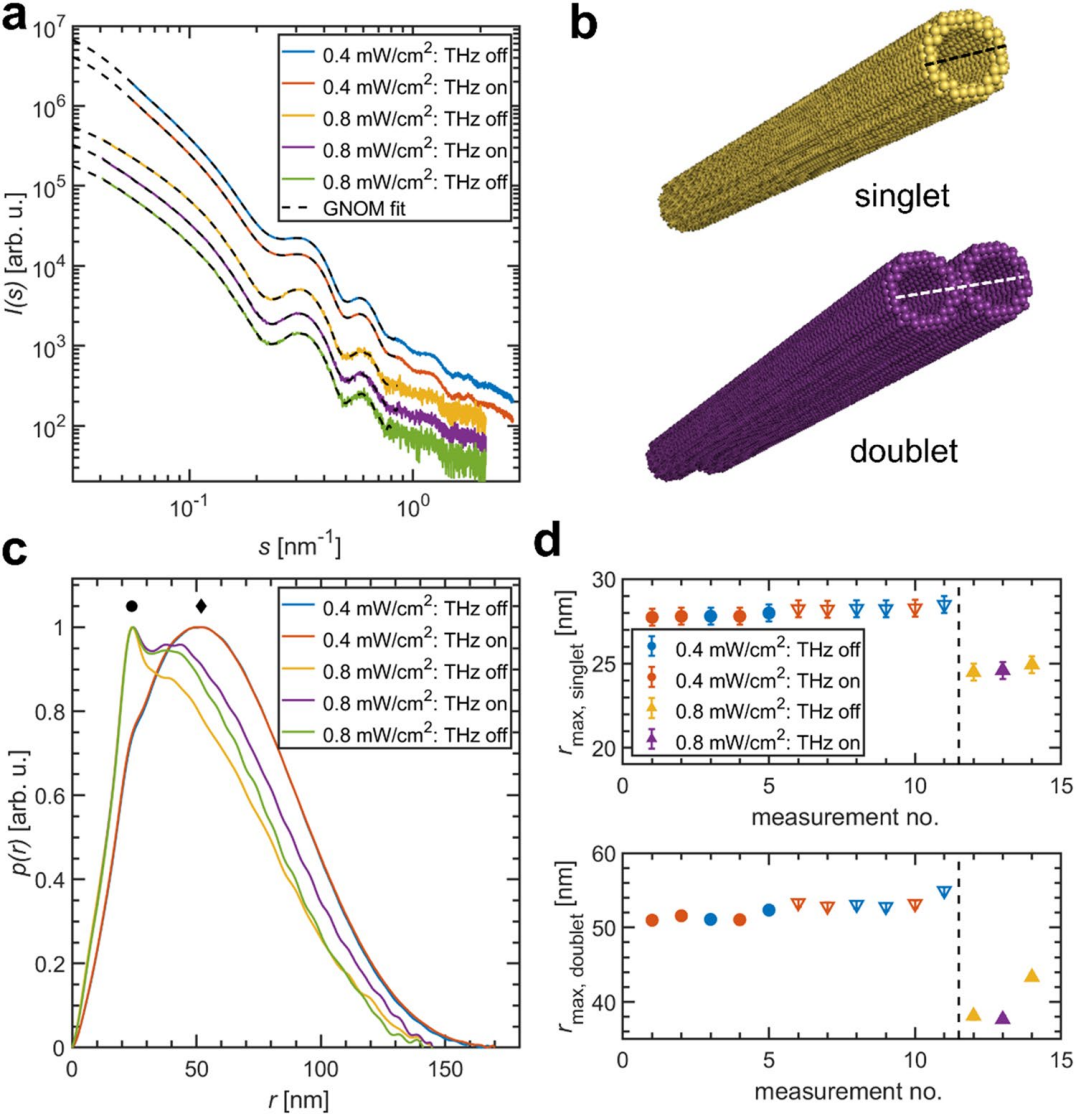
THz-SAXS measurements on microtubules. (**a**) SAXS profiles from exposed and non-exposed samples. Data have been collected in two independent beamtime sessions using different THz power densities. Dashed lines: GNOM fits. (**b**) Approximate structural models using dummy beads to compute the SAXS signal for singlet and doublets build using the ATSAS program BODIES (beads do not represent tubulin subunits). (**c**) Pair-distance distribution function p(r) computed from the SAXS curves displayed in (**a**) determined with GNOM. Characteristic features due to the structure of singlet and doublet are marked by a dot (singlet) and a diamond (doublet). (**d**) Effect of THz-exposure on the effective singlet and doublet diameter. The dashed line separates different sample preparation, e.g., different protein concentrations. Different symbols mark different MT batches.

Figure 5 presents the THz-SAXS results obtained for MT at two different THz powers collected within two separate experiments using different sample batches. The SAXS profiles of MT reflect the shape of elongated hollow cylinders^53,58–60^, with an average inner diameter *R*_i_ = 8.4 nm and a wall thickness *t* = 4.8 nm (see §SI-3for details). The sample preparation followed the complex protocols (“Methods”), and MTs in different states of bundling were present in the two experiments.

Structural information on MTs is retrieved by analysing the corresponding distance distribution functions *p*(*r*) computed by an indirect Fourier transform of the scattering curve and yielding a real-space representation of the MT structure (Fig. 5c; see “Methods” for details). Here, two characteristic features are highlighted, one maximum at *r* = 28 nm and another at *r* = 50 nm. The first maximum can be attributed to the effective cross-section diameter of the hollow singlet tubes, while the second one appears to reflect the presence of two associated MTs which are in close contact (MT doublets in Fig. 5b). In particular, the doublet contribution is lower for the second batch studied (Φ = 0.8 mW/cm^2^ data set). Due to the limited resolution of the experiment for very small angles, the total size of the MTs cannot be determined. The maximum size *D*_max_ is therefore only reflecting the accessible *s*-range and not to be mistaken with the total MT length, which can be above 1 µm^53,60^.

When exposed to THz-radiation, there are no visible differences in the SAXS curves for Φ = 0.4 mW/cm^2^ (Fig. 5a), neither on the position of the minima, reflecting the MT diameter, nor in the decay at small *s*, being sensitive to the stiffness of the rod-like protein assembly. This can be also seen when comparing the *p*(*r*) functions, which are fully overlapping. Especially the position of both maxima is unaffected (Fig. 5c), implying neither a change in the diameter nor any dissociation of doublets by THz.

For the experiment using an increased source power density, Φ = 0.8 mW/cm^2^, and a different batch, only a limited number of data points could be collected (2 × off; 1 × on). Although the *p*(*r*) functions exhibit some changes between off- and on-state, differences are also present between both ‘THz off’ data. This indicates that for MT other factors, e.g., sample inhomogeneity, rather than THz-induced conformation changes are influencing the SAXS pattern. Given the limited data, no specific THz-effect (thermal or non-thermal) can be reliably detected. It should be noted that the position of the first maximum is unaffected by THz influence, implying that also under this condition the MT diameter changes are less than 0.1 nm (Fig. 5d).

From the limited data sets collected on MTs, no indications are observed that THz radiation affects their structure in terms of size or dissociation of multiplets on the nanometre scale, which should have been visible by SAXS. Given the high symmetry of MTs, any excitation and resulting, even weak, changes should have been reflected by a change in the singlet and doublet diameters and thus in the position of the pronounced first minima of *I*(*s*). As the MT diameter is not visibly changing upon exposure, this suggests that any THz-induced structural changes are less than 0.1 nm for the given experimental parameters and low power densities, which is smaller than the first water hydration shell.

## Discussion

The aim of the combined THz-SAXS experiments was to validate the existence of THz-induced global structural changes in biological macromolecules for different sample systems under various experimental parameters. To fulfil all requirements for a combined THz-SAXS experiment, we developed a dedicated setup employing a novel microfluidic sample cell^45^. Our study reveals that THz-radiation for low nominal power densities of 6.8 mW/cm^2^ at 0.5 THz and 0.8 mW/cm^2^ from 0.1 to 5.0 THz does not lead to any large-scale structural changes larger than 0.3 nm, the size of the first water hydration layer, in the protein systems investigated. The detailed data analysis confirms that there are (a) no changes of the structure or monomer–dimer composition for BSA either for irradiated solutions or powders (see §SI-2) (maximum observed size change is 0.02 nm), and (b) no changes of the diameter and assembly for MTs (less than 0.1 nm). Below we compare our experimental conditions with those of previous studies, which suggested the presence of such thermal and non-thermal excitations and, in particular, of Fröhlich condensation.

We chose the two sample systems studied for two reasons: (1) Both systems have previously been well characterized by SAXS so that even weak structural changes can be distinguished. (2) Both systems have been proposed to be susceptible to non-thermal THz-induced changes and Fröhlich condensation. Because of the different shape morphology, both protein systems could be considered as representatives of different structural classes.

In a recent theoretical study, Zhang et al. gave an estimate on the expected power needed to excite Fröhlich condensates for MT, BSA and lysozyme^41^. Based on their calculations, BSA (and lysozyme) would be better candidates than MTs to observe Fröhlich condensation as the excitation of MTs’ longitudinal modes would require a higher pumping power. For achieving a condensate ratio of 50% for BSA, the authors considered THz-radiation of 400 µm wavelength (frequency: 0.75 THz) demanding a power of 8 W, which is more than three orders of magnitude larger than the power reached with our THz-sources. For such high THz powers, however, thermal effects are expected to dominate any non-thermal effects^13^. The estimated temperature increases for the THz sources used by us is 120 mK, assuming complete THz-absorption and thermalization in water. This is an insignificant change for the protein solutions at ambient conditions, as for instance for BSA *R*_g_ is unchanged until 40 °C^38^, ruling out thermal effects using weak THz sources.

An important parameter is the time-scale for probing the system. An exposure time in the range of *t*_exp_ = 50–200 ms was chosen for collecting single SAXS curves, and the dwell time of the flowing sample in the THz spot was ~ 0.4 s. This duration is larger than the lifetime of micro- to millisecond scale reported by Lundholm et al.^29^, who probed changes on the atomic scale, however much shorter than minutes and even hours on which other changes have been reported^22,28,30,34^. Thus, while our choice of the data collection strategy resulted in a rather low total amount of THz energy deposited into the samples, it was aimed at ensuring the collection of high quality SAXS curves within several repeats reducing X-ray induced radiation damage by sample flow.

This SAXS study is, to our knowledge, the first one that aimed to directly probe global changes of the solution structure of biological macromolecules by THz-radiation on the nanoscale. The experimental parameters covered a broad range of conditions and for none of these did we observe THz-induced structural changes of the biological macromolecules. If there are any THz-induced changes in the samples at these specific conditions, they must be small (sub-nm scale).

This proposal on the absence of strong large-scale excitations is consistent with the results from some experimental THz-based studies. In particular, in the crystallographic study on lysozyme, slight changes in the electron density of a single helix by THz-radiation were observed^29^, while for trypsin crystals an increase of the anisotropy of atomic displacements for neighbouring residues was reported^31^. The structural changes observed for lyophilized BSA powders after long-time THz-exposure have been determined only indirectly by optical spectroscopy^28^, which is only probing local protein structure.

SAXS profiles computed from the crystallographic structures of lysozyme^29^ and trypsin^31^ crystals exposed to THz-radiation revealed no visible changes in a wide angular range (see §SI-4). This shows that the non-thermal changes of the structure of proteins induced by THz irradiation may be very weak not resulting in large scale alternations.

The case of Fröhlich condensation within MT has been matter of long-time dispute, in particular because of the prominent role of MT for the cell architecture. While there have been claims on the existence on long-lived, low-frequency exited states^42,43^, other theoretical considerations suggested that the lifetime due to damping is too short to allow for collective vibrational processes^61^. A recent molecular dynamic study determined the vibrational properties of MT to be similar to those of other globular proteins and thus suggested that MT are actually unlikely candidates for Fröhlich condensates^62^. Only just now, Hough et al. reported the disassembly of MTs^34^, by using more intense THz pulses (up to Φ = 68 mW/cm^2^, peak electric field *E*_peak_ = 409 kV/cm) than in our work (Φ = 0.8 mW/cm^2^, *E*_peak_ = 18 kV/cm). After exposing MT samples to THz radiation for several minutes to 1 h, the authors find dose (and possible frequency) dependent MT disassembly, which could not be explained by heating or shockwave formation^32^, but suggest a coupling to the dynamics of the MT structure. In our present study, using lower THz densities and probing shorter time scales, we do not find any structural changes, neither on single MT (singlet) nor on the associates (doublet). In particular, we do not find any THz-response of MT or BSA on the nanometre-scale, despite their different shape morphology. Based on our findings and recent ones from others^32,34,41^, using higher power densities but still avoiding thermal heating of the samples appears as the only sufficient way to determine in which way THz-radiation might induce any non-thermal microscopic structural changes.

Summarizing, our extensive combined THz-SAXS study documents that the effect of THz radiation of 0.5 THz and 0.1–5.0 THz and of rather small nominal power densities of 6.8 mW/cm^2^ and 0.8 mW/cm^2^, respectively, on biological macromolecules appears to be below the detection limit of SAXS. There has been no apparent structural response from two protein systems expected to show THz-induced changes, BSA and MT. This sets a lower limit for future experiments on exploring the existence of long-range Fröhlich condensation or any other non-thermal effects for proteins in solution; in particular, we did not find indications for any collective domain motions which would have manifested themselves at these length scales.

## Methods

### Sample preparation

BSA, lyophilized powder, crystallized, > 98%, was purchased from Sigma-Aldrich and used without further purification. Protein samples were freshly prepared prior to the SAXS measurements. BSA powder was dissolved in a buffer of 25 mM HEPES, 50 mM NaCl, and 3% v/v glycerol, pH 7. Final concentrations were determined by UV absorption using a NanoDrop spectrophotometer.

MTs: Porcine tubulin was purified from the fresh porcine brain according to the published protocol^63^. For the measurements, the MT were polymerized from purified tubulin (7 mg/mL) by incubation at 37 °C for 10–20 min using 1 mM GTP in buffer consisting of 100 mM Pipes of pH 6.9, 1 mM MgCl_2_, 1 mM EGTA, and 1 mM dithiothreitol. For the initial measurement shown here, we have used Taxol stabilization by adding 10 μM Taxol, respectively.

### THz-setup

For the combined THz-SAXS experiments, a dedicated 3D-printable microfluidic cell was designed. In short, the cell design was chosen to allow for sample flow of small amounts of dilute, radiation-sensitive protein solutions, to be transparent for THz-radiation to excite these solutions and to allow for collection of high quality SAXS patterns. To achieve this, non-polar polystyrene (PS) was used as the cell wall material is transparent to THz-radiation. The beam paths of the THz and the X-ray beam are perpendicular to each other and to the direction of sample flow within the microfluidic channel. An asymmetric profile of this channel (thickness *d*_THz_ = 0.25 mm (THz beam path) × *d*_X-ray_ = 2 mm (X-ray beam path) accounts for the different absorption length of THz and X-ray radiation, respectively, and therefore yields optimum excitation and scattering conditions. The THz absorption length for PS is α_PS_ = 2.21 cm^−1^ and that of water α_H2O_ = 220 cm^−1^^49^, which results in a ~ 72% transmission of the first PS window, while the THz radiation is close to be completely absorbed within the thin channel when filled with protein solution (see §SI-5). The possibility of measuring the THz-transmission through this cell ensures a proper alignment of the microfluidic channel to the X-ray beam such that the same portion of the sample is irradiated by THz and probed by SAXS. Further details on the microfluidic cell can be found in Ref.^45^.

Two THz-sources with different parameters were used to study the level of excitation:

Source I generates THz radiation by an amplifier/frequency multiplier chain (X32 stage AMC, Virginia Diodes Inc., Charlottesville, VA, USA), which is driven by a MG3692c microwave signal generator (Anritsu)^31^. The input microwave radiation was set to 15.625 GHz (10 mW), which resulted in continuous wave (cw) 0.5 THz radiation of 1 mW power. A diagonal horn antenna (WR-2.2) was positioned approximately at 0.5 cm from the microfluidic cell. Under the assumption of a Gaussian beam profile, the THz spot size diameter *d* was approximately *d* = 4 mm. This estimate assumes a waist radius of 1.3 mm and that the beam originates from approximately 1/3 inside the antenna.

Source II is a TeraFlash THz-setup (TOPTICA Photonics AG, Graefelfing, Germany), which operates in the time-domain^45^. THz pulses are generated with an average power of *P*_aver_ = 30 µW (average electric field *E*_aver_ = 110 V/cm), a peak power of *P*_peak_ = 0.4 W at 0.4 THz (peak electric field *E*_peak_ = 13 kV/cm) and a THz pulse width of 600 fs at a repetition rate of 100 MHz, as specified by the company. These THz pulses are split into a probe and reference pulses. The probe pulse is first guided through a set of focussing parabolic mirrors with a focal length of 25.4 mm to a spot size diameter of *d* = 3 mm on the sample. The focused beam is then forwarded to a receiver, composed of an InGaAs/InP photoconductive switch with a 25 µm dipole antenna, where it interferes with the reference pulse. A subsequent Fourier transformation provides the absorption spectrum. For THz-SAXS measurements, the microfluidic cell was placed into the focal point created by the THz-mirrors and the receiver is used only as an intensity detector for alignment of the THz optics and the cell. During the SAXS experiment, the receiver was replaced by a second emitter to excite the protein systems from two directions for a more homogeneous illumination with a higher power (*P*_aver_ = 60 µW, *E*_aver_ = 150 V/cm; *P*_peak_ = 0.8 W, *E*_peak_ = 18 kV/cm).

The power density of the THz sources is given by Φ = *P*_aver_/(π(*d*/2)^2^), in which *P*_aver_ is the average power and *d* the focal spot size diameter.

The maximum temperature rise can be estimated assuming full THz absorption and thermalization by water. For a power of *P* = 1 mW, a flow rate of $\dot{V}$ = 2 µL/s, density of water ϱ = 1 kg/L, specific heat capacity of water *C* = 4.2 kJ/kg^/^K, the maximum achievable temperature increase under ambient conditions is Δ*T* = *P*/($\dot{V}\mathrm{\varrho } C$) = 120 mK. Due to absorption by cell window and other effects, the real increase will be even smaller.

### SAXS measurements

SAXS measurements were performed at the BioSAXS beamline P12, EMBL/PETRA III, Hamburg, Germany^64^, within several experimental sessions. Table 1 gives an overview of the relevant experimental parameters.

**Table 1:** Overview of the experimental parameters of the different THz-SAXS beamtimes.

Date	Dec 2018	Aug 2019	Oct 2019	Aug 2020
THz source	Source II	Source II	Source I	Source II
Frequency [THz]	0.1–6.0	0.1–6.0	0.5	0.1–6.0
Cw/pulsed	Pulsed: 100 MHz	Pulsed:100 MHz	Cw	Pulsed: 100 MHz
Power (average) [mW]	0.03	0.06	1.0	0.06
Power density [mW/cm^2^]	0.4	0.8	6.5	0.8
Spot size diameter (free) [mm]	3	3	4	3
Spot size* (on sample) [mm]	2	2	2	2
Deposited energy** [µJ]	5	4	75	10
**SAXS**				
X-ray energy [keV]/wavelength [nm]	10/0.124	10/0.124	10/0.124	10/0.124
Sample-detector distance [m]	3.0	4.0	3.0	3.0
Detector	PILATUS 6 M	PILATUS 6 M	PILATUS 6 M	PILATUS 6 M
Exposure time [ms]	100	100, 200	200	50
Number of frames	50	50	250	50
Samples	Microtubules	Microtubules/BSA	BSA	BSA
Concentration [mg/mL]	3.5	3.5/4.4–5.3	5.8	2.5, 10.9
Sample volume [µl]	120	120	120	120
Flow rate [µL/s]	2	5	2	2
Dwell time (in THz beam) [s]	0.4	0.2	0.4	0.4
THz status during SAXS	On/off	On/off	On/off/alternating	On/off

*Defined by sample cell aperture of *w* = 2 mm.

**Taking into account the dwell time of the sample in the THz beam and the transmission of the PS window.

For each experiment, the entire THz-setup was placed within the air gap between the evacuated X-ray beam path sealed by two Kapton windows. This gap was minimized to reduce the parasitic scattering and absorption from air. The X-ray beam was cut down to about 100 × 200 μm^2^ using tungsten slits to fit into the narrow channel of the microfluidic cell and to reduce the parasitic background signal from the polystyrene matrix. The resulting photon flux was ~ 10^12^ ph/s. Parasitic scattering from the beam-defining slits was reduced using a second pair of scatterless slits close to the sample position. Two-dimensional scattering patterns were recorded using a PILATUS 6 M detector (DECTRIS, Villigen, Switzerland). For the individual beamtimes, different numbers of 2D frames and exposure times were used (see Table 1).

For the precise cell alignment, a remotely controlled hexapod (HXP100-MECA, Newport, Irvine, CA, USA) was used. To avoid radiation damage, the samples were moved continuously by using a remotely controlled syringe pump (neMESYS, Cetoni GmbH, Korbussen, Germany) in all experiments. For the measurements of protein and buffer solutions, polyethylene tubing-loops filled with buffer and sample were connected between the pump and the cell. The total sample volume used for filling the tubing was of 1 mL, from which a volume *V* = 120 µL was used per measurement, that was moved at defined flow rates in the range of $\dot{V}$ = 2–5 µL/s. This results in a dwell time of the sample in the THz-beam passing into the microfluidic cell of *t*_dwell_ = $w \bullet ({\dot{V }{d}_{\mathrm{THz}} {d}_{\mathrm{X}-\mathrm{ray}})}^{-1}$ = 0.2–0.4 s. Here, *w* is the THz beam size within the sample of *w* = 2 mm which is defined by the sample cell geometry. To prevent the buffer and protein solution from uncontrolled mixing, small air inclusions between the two liquids served as spacers inside the tubing during sample loading.

### Data collection

SAXS patterns were collected from protein and buffer solutions without (‘THz off’) and with full (‘THz on’) THz irradiation. Due to the loading procedure, first a sequence of buffers for both THz states was collected and subsequently the signals from the protein solutions. This data collection cycle was repeated several times, using different batches of protein solution within a single beam time.

In addition, for THz source I, the terahertz radiation source was also operated alternately. Such modulations allow one to reduce the systematic differences associated with possible thermal effects. A DG645 pulse (delay) generator (Stanford Research Systems) was triggered on the raising edge of the output EN OUT signal of the PILATUS 6 M detector. Upon triggering, the pulse generator was programmed to send a pulse to the terahertz radiation source (‘THz on’ period). Simultaneously, the pulse generator was set to ignore subsequent triggers for 200 ms. Consequently, every odd numbered frame was in the terahertz irradiated state (‘alt. THz on’), and every even numbered frame in the terahertz off state (‘alt. THz off’). Such a collection scheme was not possible for pulsed THz source II.

Due to the different ways of THz exposure, for source I, a long collection of 250 frames each 0.2 s was performed, in order to test for any variation within the alternating THz exposure, while for source II a short collection of 50 frames was conducted.

### Data analysis

Two-dimensional SAXS patterns were azimuthally integrated, after masking out beam stop shadow and inter-module segments, and calibration the angular axis using the P12 beamline SASFLOW pipeline^65^, which yields 1D SAXS profiles. As the buffer data—including the background scattering contribution of the sample cell and beamline—did not show any effect of THz radiation, these curves were averaged and subtracted from the protein scattering yielding the net signal *I*(*s*).

Given the different data collection strategies for the two THz sources, the data analysis was also made in different ways. For the long series recorded for source I, the averaged buffer curve was subtracted from each individual SAXS profile of the protein solution for the further analysis. For source II, with either THz on or off status, the individual frames were averaged using the P12 SASFLOW pipeline before buffer subtraction.

For the BSA data collected during the different beamtimes, relevant parameters were extracted to probe the effect of THz-irradiation on the protein structure and oligomeric state using the programs from the ATSAS software package^66^ (https://www.embl-hamburg.de/biosaxs/download.html; version 3.0.3).

The radius of gyration *R*_g_ was determined following the Guinier approximation for small *s*^35,47^,

$$I\left(s\right)\approx I\left(0\right)\,\,\mathrm{exp}\left(-\frac{{s}^{2}{R}_{\mathrm{g}}^{2}}{3}\right),$$

using PRIMUS^67^ of the ATSAS package.

The volume fractions of monomers ${\nu }_{\mathrm{m}}$ were determined by fitting the scattering from high resolution structures of BSA monomer and dimer (RCSB PDB file 3V03)^68^ to the experimental SAXS curves using the ATSAS program OLIGOMER^67^ following the relation:

$$I\left(s\right)= {\nu }_{\mathrm{m}}{I}_{\mathrm{m}}\left(s\right)+ {\nu }_{\mathrm{d}}{I}_{\mathrm{d}}\left(s\right)$$

wherein the indices ‘m’ and ‘d’ refer to monomer and dimer, respectively. As the entire SAXS profile is used, the monomer fraction serves as a parameter sensitive to the full curve and thus also to changes at intermediate length scales.

For the long data collection with THz source I, the discrepancy between two adjacent single frames *n* and *n* + 1 is computed as

$${\chi }_{n,n+1}^{2}= \frac{1}{N+1}\sum_{j=1}^{N}{\left(\frac{{I}_{n}\left(s\right)-{I}_{n+1}\left(s\right)}{\sqrt{{\sigma }_{n}^{2}+{\sigma }_{n+1}^{2} }}\right)}^{2}$$

where *N* denotes the number of data points of a curve, and $\sigma$ the experimental error.

For the data collection with THz source I, normalized histograms for the radius of gyration, the monomer volume fraction, and the pair discrepancy were generated. The profiles were fitted using Gaussian functions, respectively.

For microtubules, which exhibit long hollow shapes completely different from BSA, another approach was used: The pair-distance distribution *p(r)* was computed using GNOM^35,69^, and the positions of its characteristic features were extracted. The pair-distance distribution function is related to the scattering curve via a Fourier transformation and reads

$$p\left(r\right)=\frac{1}{2{\pi }^{2}}{\int }_{0}^{\infty }I(s)\,sr\,\mathrm{sin}\left(sr\right)ds$$

wherein *r* denotes the spatial coordinate. The *p*(*r*) function differs from zero for *r* < *d*_max_, the maximum particle size.

Care was taken to rule out effects of radiation damage which would affect the data interpretation, in particular, for the expected very weak THz-induced changes. All samples were continuously flowed while being exposed to the X-ray beam. Data collection was repeated several times, and the data sets exhibiting indications of beam-induced changes (e.g., increase in *R*_g_ within a collection series or drop of intensity due to (micro-)bubble formation)^70,71^ were fully excluded from the analysis. By this we ensured that only data free of artefacts were used for analysis and interpretation.

Theoretical SAXS curves have been computed from high-resolution crystallographic data under THz irradiation using the program CRYSOL^72^. For this, the following structures were used from the RSC PBD: Hen egg white lysozyme^29^: 5APC (12 mW @0.4 THz, on), 5APD (THz off), 5APE (reference odd), 5APF (reference even); Bovine trypsin^31^: 6SUX, 6SV8, 6SVB, 6SVG, 6SVJ (1mW @ 0.5 THz, on); 6SV0, 6SV6, 6SV9, 6SVD, 6SVI (THz off); 6SVR, 6SVV, 6SVX, 6SW0 (reference, odd); 6SVU, 6SVN, 6SVD, 6SVZ (reference, even).

## Supplementary Information

Supplementary Information.
