# Supplementary material for: Probing the existence of non-thermal Terahertz radiation induced changes of the protein solution structure
Source: Sci Rep. 2021 Nov 16;11:22311. doi: 10.1038/s41598-021-01774-6 (PMC8595702; doi:10.1038/s41598-021-01774-6)
Supplement: Supplementary file 1 — Supplementary Information. [file 41598_2021_1774_MOESM1_ESM.pdf]

Supplementary Information to

Probing the existence of non-thermal Terahertz radiation induced changes of the protein solution structure

Martin A. Schroer[1,*], Siawosch Schewa[2], Andrey Yu. Gruzinov[1], Christian Rönnau[3], Janine Mia Lahey-Rudolph[2], Clement E. Blanchet[1], Till Zickmantel[3], Young-Hwa Song[3], Dmitri I. Svergun[1], Manfred Roessle[2]

[1] European Molecular Biology Laboratory (EMBL), Hamburg Outstation c/o DESY, Notkestr. 85, 22607 Hamburg, Germany.

[2] University of Applied Sciences Luebeck, Moenkhofer Weg 239, 23562 Luebeck, Germany.

[3]Institute of Physics, University of Luebeck, Ratzeburger Allee 160, 23562 Luebeck, Germany.

*corresponding author: martin.schroer@uni-due.de; present address: Nanoparticle Process Technology, University of Duisburg-Essen, Lotharstr. 1, 47057 Duisburg, Germany.

§SI-1: THz-SAXS on BSA solutions – Additional parameters

Identical observations as for the radius of gyration are present for the monomer volume fraction ν_m determined from the SAXS data of BSA by the OLGIOMER analysis (SI-Fig. 1-3). This approach is sensitive to the whole SAXS profile, ensuring that the full curve is analysed. But even without assuming any model, the smallness of any existing THz-effects can be seen when looking on the discrepancy $\chi^2_{n,n+1}$between two adjacent SAXS frames. For alternating THz-exposure, the average $< \chi^2_{n,n+1} >$ is only slightly larger than for the off and on conditions (SI-Fig. 3), which are basically equal to unity, revealing the absence of any systematic changes. Given the overall very weak variations, a clear existence of any THz-induced changes cannot be made.

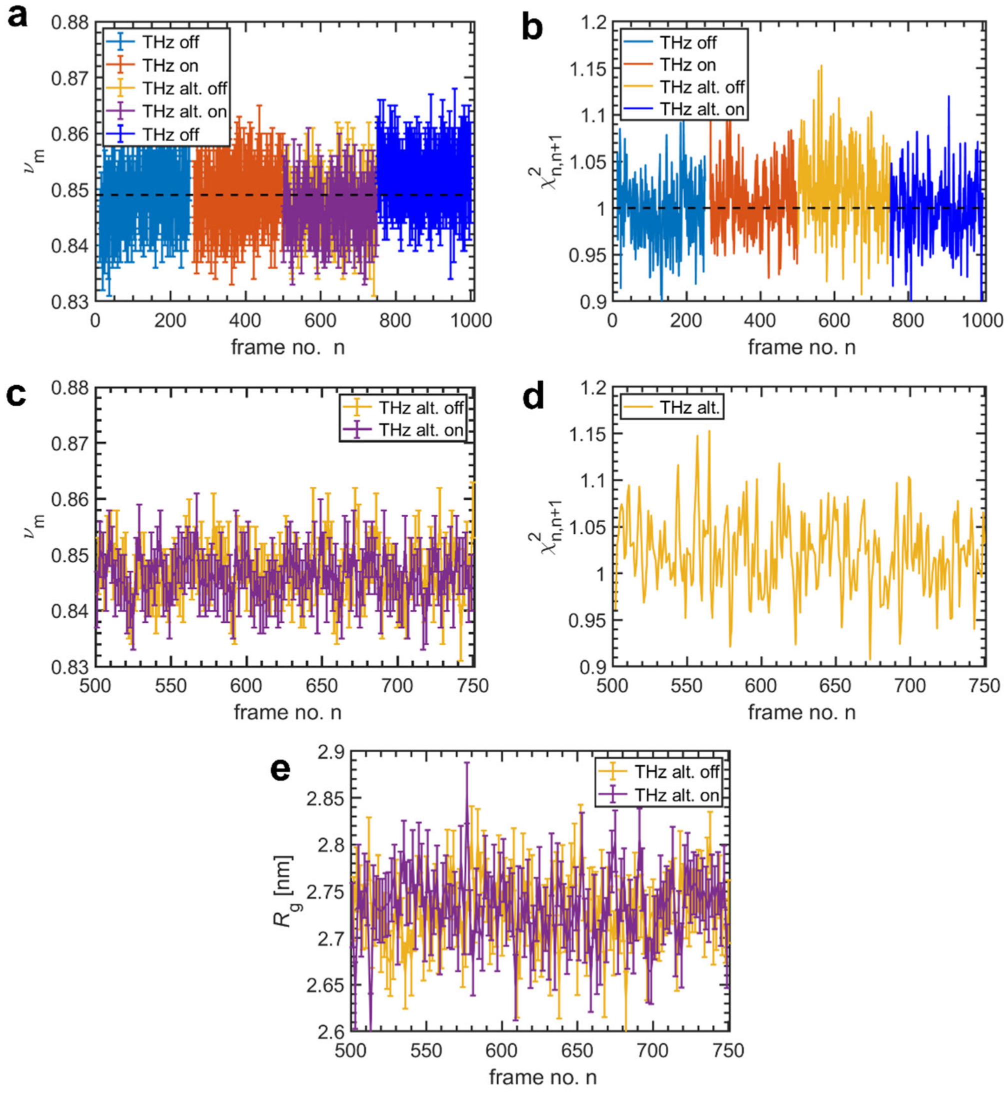

SI-Figure 1: Extracted parameters for SAXS data collection of a BSA solution (c = 5.8 mg/mL) exposed to Φ = 6.5 mW/cm^2 at 0.5 THz (THz source 1). a) Monomer fraction ν_m for a collection of THz off, THz on, THz alternating and THz off again, extracted for each of 250 individual SAXS frames. b) Discrepancy $\chi^2_{n,n+1}$ for two adjacent frames. Enlargement of the parameter traces for alternating THz-exposure: c) ν_m, d) $\chi^2_{n,n+1}$ and e) R_g.

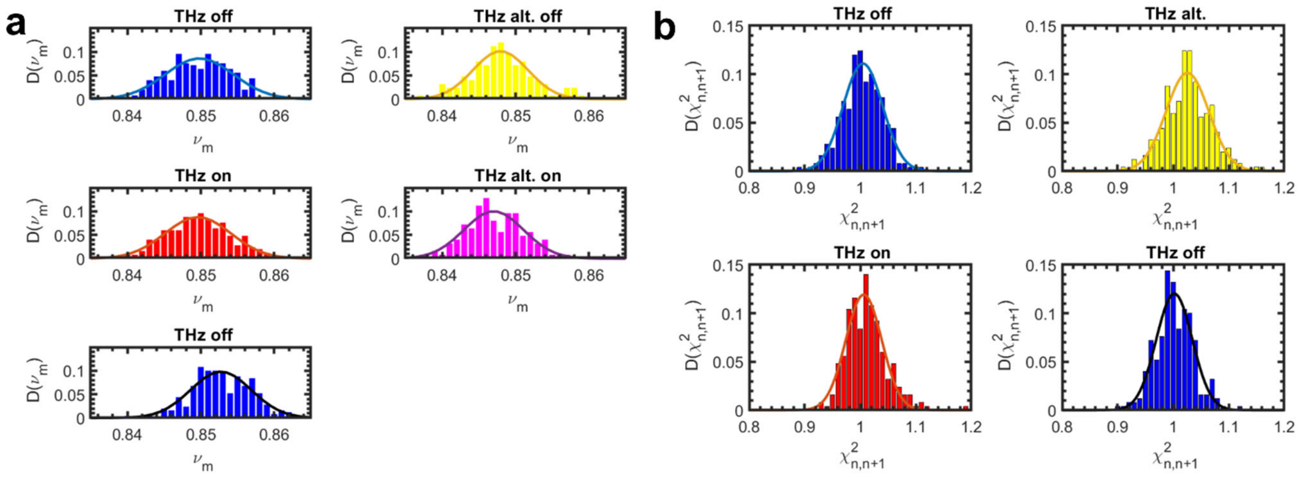

SI-Figure 2: Normalized distributions of a) monomer volume fraction ν_m and b) pair discrepancy $\chi^2_{n,n+1}$ for THz-exposure with source I. As $\chi^2_{n,n+1}$ is computed for two adjacent frames, there is only one distribution for alternating THz (THz alt.). Solid lines are Gaussian fits to the histogram.

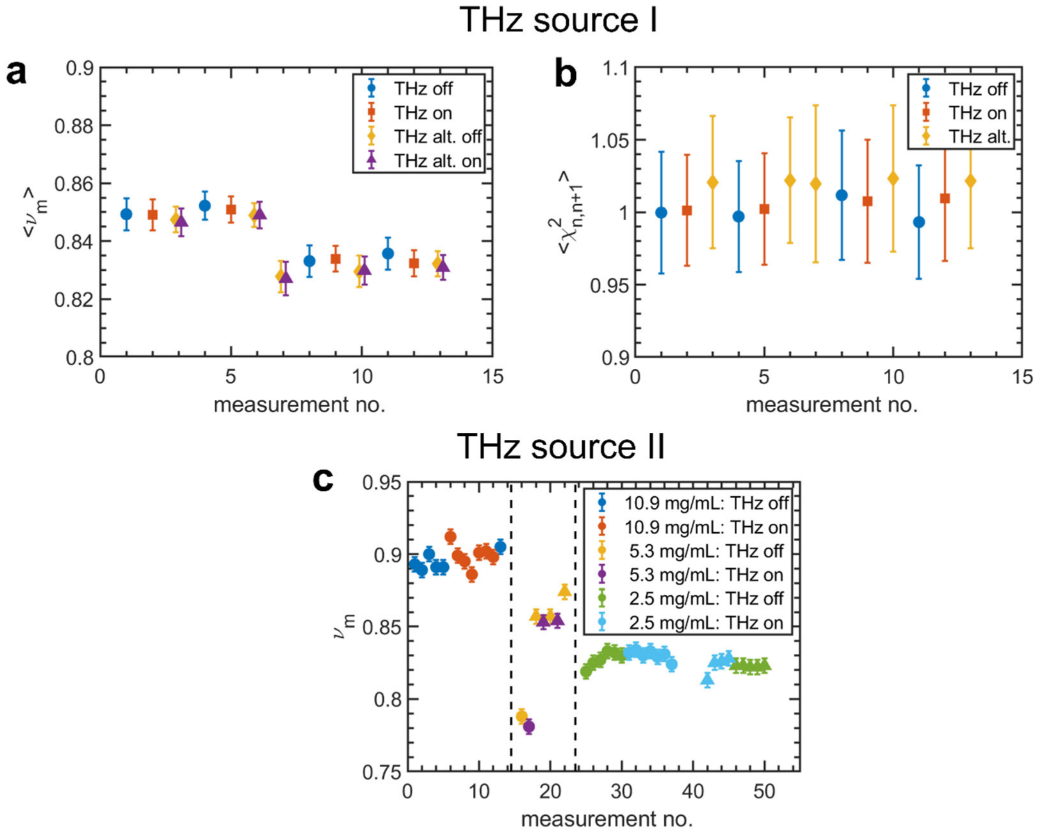

SI-Figure 3: Structural parameters for several repeats. For THz source I: a) Averaged monomer volume fraction $<\nu_m>$ and b) average discrepancy $<\chi^2_{n,n+1}>$ as determined from the corresponding distribution functions in Si-Fig.2. For THz source II: c) Monomer fraction using different BSA concentrations. Different batches are marked by different symbols.

§SI-2 Pre-exposed lyophilized BSA powders

In another attempt to explore if THz-radiation can induce any structural changes in BSA that can be detected by SAXS, lyophilized BSA powder was exposed to THz radiation for ~24 hours using THz source II. With this approach, we follow the study of Cherkasova et al.[1], who irradiated BSA powder with radiation of 3.6 THz and 10 mW and reported changes in the UV absorption and circular dichroism spectra, and related these to conformational changes of the protein. Our THz setup II covers in particular 3.6 THz, the frequency used in that study.

Two aliquots of the same batch of lyophilized BSA powder were filled into standard polypropylene tubes. One of these BSA powders was exposed at ambient temperature for ~24 h to THz radiation of source II within its tube, which is transparent to THz [2]. The other specimen was stored similarly but non-exposed to THz. Afterwards, both samples were shipped to the beamline, where the powders were dissolved in HEPES buffer immediately before the data collection. Standard batch mode measurements were performed within an in-vacuum quartz capillary using the robotic P12 sample changer with continuous flow[3]. Before and after the protein solutions, the buffer solution was measured. In addition, a third, freshly prepared BSA sample was measured for comparison. For all three samples, several SAXS data collections were performed within a time range of ~150 min. For each collection, 40 frames of 100 ms exposure time were taken. Similar to the previous spectroscopic study, SAXS patterns were taken at different time points after dissolving the powders.

The resulting SAXS curves – similar to those from the combined THz-SAXS measurements - do not reveal any significant structural changes (SI-Fig. 4a). While a weak but systematic decrease of the radius of gyration (SI-Fig. 4b) and an increase of the monomer fraction (SI-Fig. 4c) can be detected for both samples, which we attribute to a dissociation of dimers after mixing, the difference between exposed and non-exposed samples are neglectable (SI-Fig. 4d). The difference to the third reference BSA sample is effectively larger than those between the samples exposed and non-exposed to THz.

It should be noted that due the lower scattering background of the sample changer setup, SAXS curves up to larger scattering angles can be determined. As these can be all reasonably well fitted as a monomer-dimer mixture, also significant THz-induced changes in the wide-angle scattering part of the curves can be excluded. There have been also no effects due to storing both samples at ambient temperature for the time of THz-exposure. As the previously mentioned study actually reported on changes in the solution structure of BSA after THz exposure of the powder[1], our results reveal that any such effects appear to be highly localized and not affecting the protein structure on any length scale accessible by SAXS in our experiment.

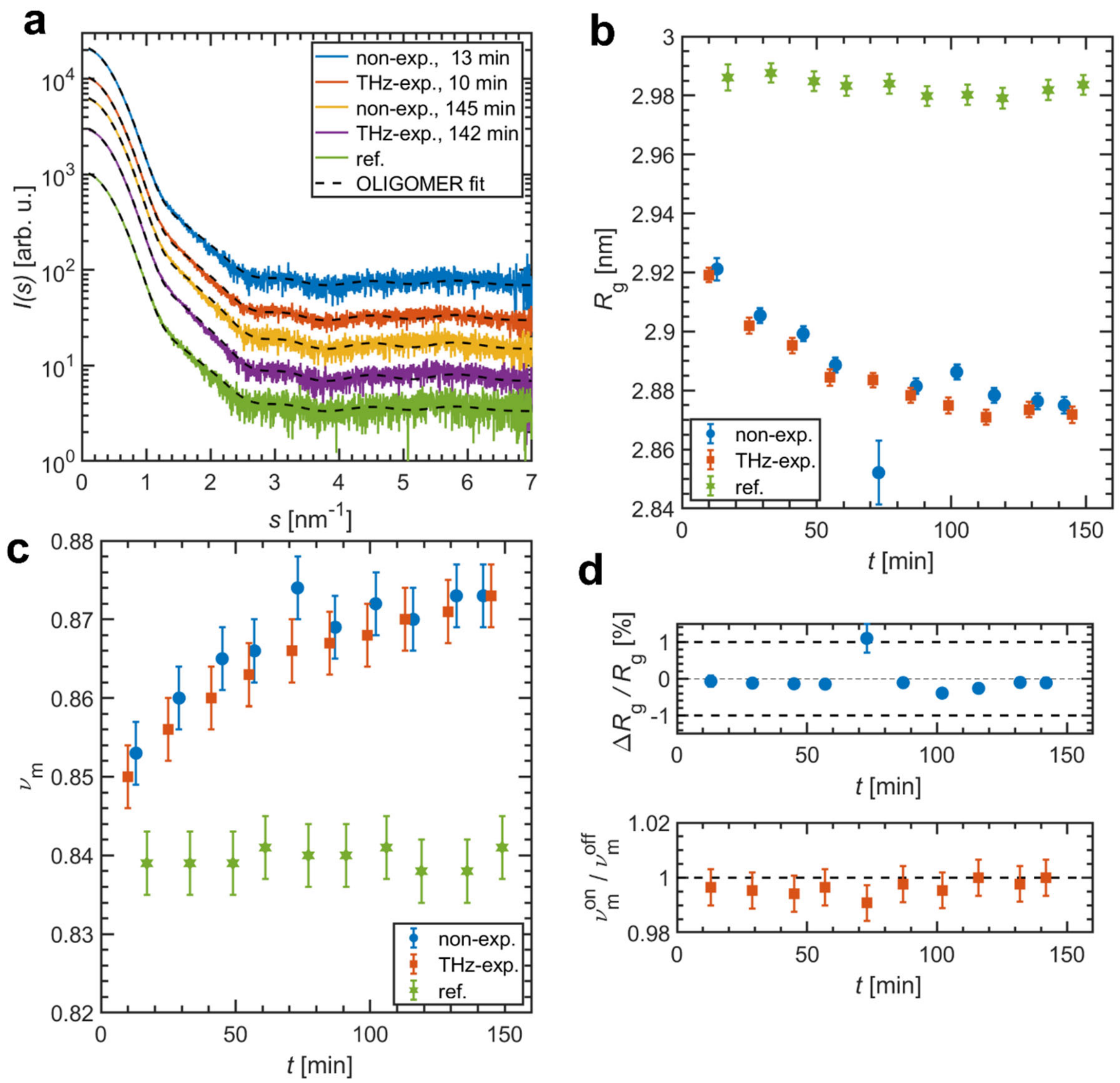

SI-Figure 4: Effect of long-time THz-exposure to lyophilized BSA powder. a) SAXS profiles of BSA from powders exposed (THz-exp.) and non-exposed (non-exp.) directly after dissolving and ~140 min later, as well as a reference sample (ref.). Protein concentration: c = 2.7 – 3.1 mg/mL. Dashed lines: OLIGOMER fits. b) Radius of gyration and c) monomer fraction as a function of time after dissolving. d) Deviation parameter $\Delta R_g/R_g$ *and monomer ratio* v_m^{on}/v_m^{off} *as a function of time. We attribute the systematic change with time to the dissociation of aggregates, which were not removed by centrifugation. The reference sample was prepared previously and centrifuged.*

§SI-3 Analysis of the microtubule (MT) structure

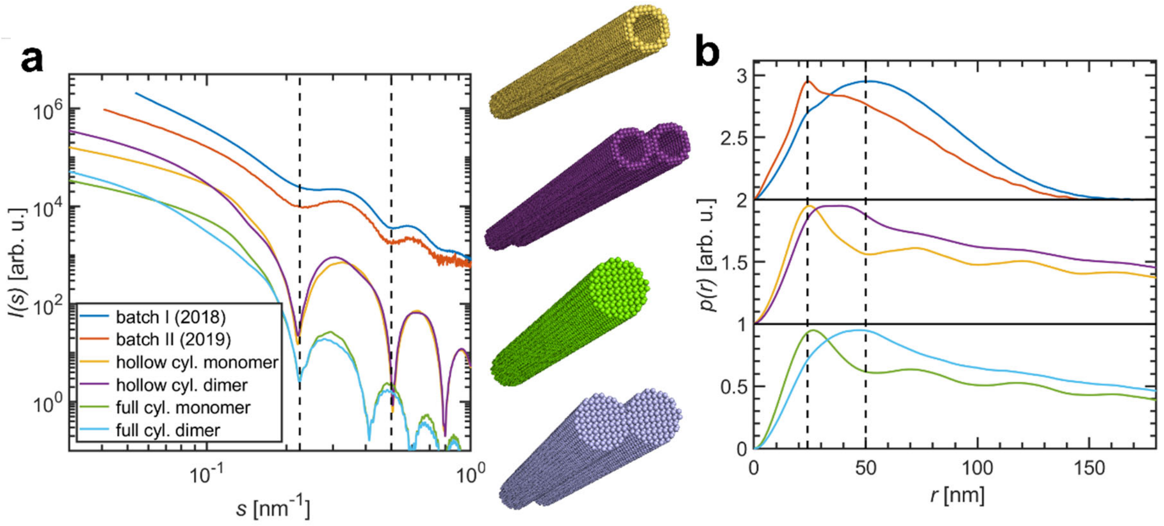

SI-Figure 5: Analysing the microtubule structure. a) Experimental and theoretical SAXS profiles for MT and different cylinders: Hollow cylinder, hollow cylinder dimer, full cylinder, full cylinder dimer. The position of the first and second minima of the experimental data are marked. b) Corresponding distance distribution functions (p(r)). The positions of the two maxima are marked, which can be attributed to the cylinder diameter and the effective dimer cross-section.

In order to understand the SAXS profiles and the corresponding $p(r)$ functions of the two batches of microtubules (MT), two sets of models were created using the ATSAS program BODIES: Single long cylinders and long hollow cylinders as well as two touching cylinders of each type. The theoretical SAXS profiles are shown in SI-Fig. 5. The height of all cylinders was arbitrarily chosen to h = 400 nm, as, due to the limited resolution of the experimental data at small angles, the total size cannot be resolved, and is not important for the following considerations.

While a full cylinder of radius R = 16.5 nm can describe the position of the first minimum for the batch II data, it fails to match the second minima. A better description is achieved for the hollow cylinder model of inner radius R_i = 8.4 nm and outer radius R_o = 13.2 nm. Note that the wall thickness $t = R_o - R_i$ = 4.8 nm is similar to the value reported in previous studies[4, 5]. The smearing of the experimental data is due to the size polydispersity of MT, which was also reported previously. Because of this, we limited our analysis to a qualitative description of the SAXS profiles, which is feasible for understanding the structural features of the $p(r)$ function.

For the SAXS profiles of two touching (hollow) cylinders, the position of the minima is unaffected but an additional modulation of the curves can be seen at smaller angles (s < 0.2 nm^{-1}). This contribution is significant when looking on the corresponding $p(r)$ functions. For both types of single cylinders, a clear maximum can be seen for r = 20 – 30 nm, whose actual position depends on the filling (full vs hollow),

and is similar to the cylinder diameter. The weak modulations at larger distances are attributed to artefacts due to the indirect Fourier transform from the modelled curves and are not further considered.

For the pair-distance distribution of the two touching cylinders (cylinder dimers), however, the maximum is much broader and shifted to larger distances, reflecting the effective dimer cross-section. The contribution of the cylinder diameter has nearly disappeared and is only implied by a weak shoulder in case of the full cylinder diameter.

Comparing the $p(r)$ functions from the experimental data with the modelled curves suggests, that for both MT batches, at least two types of MT species are present in solution, MT monomers and MT dimers, whose composition changes between the batches.

§SI-4 Simulation of SAXS curves from crystallographic structures under THz-irradiation

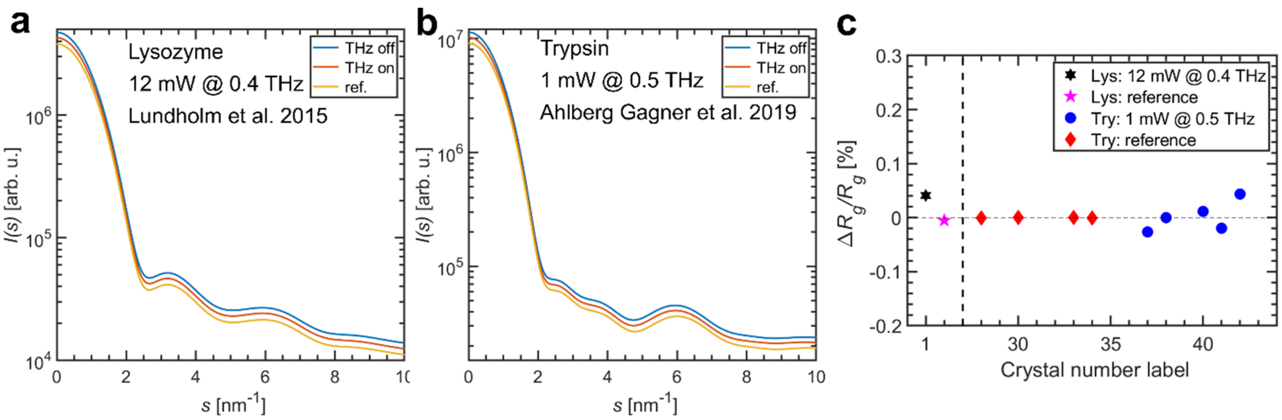

SI-Figure 6: Computed SAXS profiles from high-resolution crystallographic structures for different THz-exposure. a) Hen egg white lysozyme crystals exposed to 12 mW @ 0.4 THz (Ref. 6), b) Bovine Trypsin crystals exposed to 1 mW @ 0.5 THz (Ref. 7). SAXS curves were computed using CRYSOL and shifted for clarity. c) Relative changes $\Delta R_g/R_g$ upon THz-exposure for lysozyme (Lys) and different trypsin (Try) crystals. Reference data refer to crystal samples not exposed to THz-radiation.

In order to compare the experimental SAXS curves for proteins in solutions from the present study with the results from previous X-ray crystallography experiments of protein crystals exposed to THz-radiation[6, 7], theoretical small angle scattering profiles of the crystallographic structures were computed using the ATSAS program CRYSOL.

SI-Fig. 6a) shows the computed SAXS profiles of lysozyme crystals alternatingly irradiated with THz-radiation in the non-exposed state (THz off) and when exposed to 12 mW@ 0.4 THz (THz on) as well as of a reference crystal that was not exposed at all.

While a detailed analysis of the crystallographic data indicates slight changes in the electron density of a single helix upon THz-radiation[6], the theoretical SAXS curves do not show any indication of a change of protein structure. The same conclusion is drawn for crystal data from trypsin exposed to 1 mW@ 0.5 THz (Fig. 6b), for which an increase of the anisotropy of atomic displacements for neighbouring residues was reported[7].

In terms of the relative change of the radius of gyration for both proteins (Fig. 6c), alternating THz-exposure results in more changes than for the reference samples, however, the effect of these changes is less than 0.1%. This is even weaker than the effect observed for the experimental SAXS curves of BSA. Despite the close packing in the crystal, and the absence of large changes, an influence of proteins in solution may be possible.

§SI-5 THz spectra from the microfluidic cell

The microfluidic cell was designed for combined THz-SAXS measurements[2]. In combination with THz source II, it is possible to determine THz spectra from aqueous solutions. In particular, it allows determining the THz transmission. SI-Fig. 7 shows the time-domain THz signal from source II (without cell), from the empty cell and from the cell filled with buffer and BSA solution. When passing the cell, the top-to-bottom intensity drops from ~ 200 nA to ~ 90 nA, which is close to the expected transmission of ~ 50% of the two polystyrene windows of a total thickness of 3 mm. When filling the cell with an aqueous solution (either buffer or BSA solution), the transmitted intensity significantly drops (< 3 nA), revealing strong THz absorption of the solution. Thus, it is assured that the power of THz laser is mainly deposited in the sample volume.

This strong difference of THz absorption was also utilized for the alignment of the THz cell with respect to the X-ray beam. To ensure that the sample was completely illuminated, the transmitted signal was recorded. Positioning the cell with filled channel and minimizing the transmitted signal showed that the sample was properly exposed to the THz beam and thus the maximum power deposited.

Fig. SI-7c depicts the THz-spectra from the source and empty cell as well as from the cell filled with buffer and BSA solution. The microfluidic cell does not show any prominent absorption lines in the spectral range. Sharp absorption lines present with and without the cell stem from THz absorption by water vapor[2]. Due to high absorption and the resulting weak signal-to-noise ratio of the solutions, no differences between buffer and BSA can be distinguished.

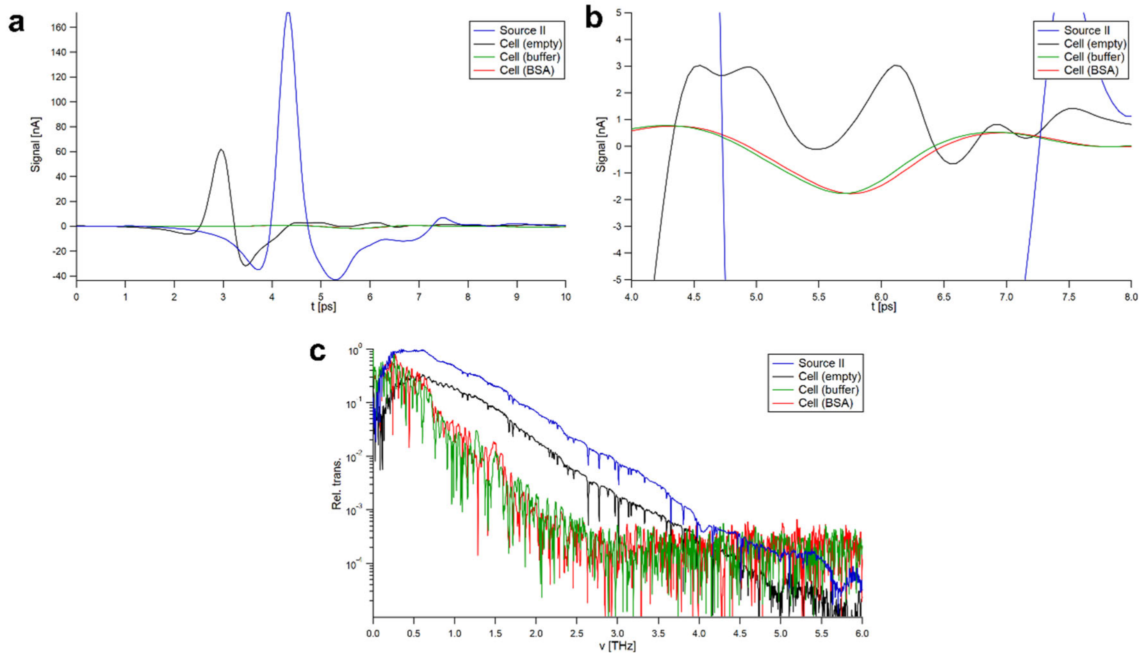

SI-Fig. 7. THz-spectra collected with THz source II using the microfluidic cell. a) Time-domain THz signal recorded for source II in absence of the cell, the empty cell, the cell filled with buffer and with a 5 mg/mL BSA solution. b) Enlargement of the signal for buffer and BSA. There are no notable differences between buffer and BSA solution detectable. c) THz spectra of the source, from the empty cell and the cell filled with buffer and BSA solution.

References

1. Cherkasova, O. P., Fedorov, V. I., Nemova, E. F. & Pogodin, A. S. Influence of terahertz laser radiation on the spectral characteristics and functional properties of albumin. *Opt. Spectrosc.* **107**, 534 (2009).
2. Schewa, S. *et al*. A THz transparent 3D printed microfluidic cell for small angle x-ray scattering. *Rev. Sci. Instrum.* **91**, 084101. (2020).
3. Round, A. *et al.* BioSAXS Sample Changer: a robotic sample changer for rapid and reliable high-throughput X-ray solution scattering experiments. *Acta Cryst. D* **71**, 67-75 (2015).
4. Choi, M. C. *et al*. Human microtubule-associated-protein tau regulates the number of protofilaments in microtubules: a synchrotron x-ray scattering study. *Biophys. J.* **97**, 519-527 (2009).
5. Chung, P. J. *et al*. Tau mediates microtubule bundle architectures mimicking fascicles of microtubules found in the axon initial segment. *Nat. Commun.* **7**, 12278 (2016).
6. Lundholm, I. V. *et al*. Terahertz radiation induces non-thermal structural changes associated with Fröhlich condensation in a protein crystal. *Struc. Dyn.* **2**, 054702 (2015).

7. Gagnér, V. A *et al*. Clustering of atomic displacement parameters in bovine trypsin reveals a distributed lattice of atoms with shared chemical properties. *Sci. Rep.* **9**, 19281. (2019).
